# Impedance Sliding-Mode Control Based on Stiffness Scheduling for Rehabilitation Robot Systems

**DOI:** 10.34133/cbsystems.0099

**Published:** 2024-06-01

**Authors:** Kexin Hu, Zhongjing Ma, Suli Zou, Jian Li, Haoran Ding

**Affiliations:** ^1^ School of Automation, Beijing Institute of Technology, Beijing, China.; ^2^ University College London, London, UK.

## Abstract

Rehabilitation robots can reproduce the rehabilitation movements of therapists by designed rehabilitation robot control methods to achieve the goal of training the patients’ motion abilities. This paper proposes an impedance sliding-mode control method based on stiffness-scheduled law for the rehabilitation robot, which can be applied to rehabilitation training with both active and passive modes. A free-model-based sliding-mode control strategy is developed to avoid model dependence and reduce the system uncertainty caused by limb shaking. Additionally, the stiffness scheduling rule automatically regulates the impedance parameter of the rehabilitation robot based on the force exerted by the patient on the robot such that the rehabilitation training caters to the patient’s health condition. The proposed method is compared with the fixed stiffness and variable stiffness impedance methods, and the superiority of the proposed method is proved. Rehabilitation training experiments on an actual rehabilitation robot are provided to demonstrate the feasibility and stability of the proposed method.

## Introduction

In recent years, human–robot interaction has received extensive attention, which has promoted the research and development of human–robot interaction devices such as service robots, surgical robots, and rehabilitation robots [1–2]. Among them, rehabilitation robots can reproduce the rehabilitation movements of therapists by the designed rehabilitation robot control methods to achieve the goal of training the patients’ motion abilities [3–5]. This kind of physical human–robot interaction has high requirements for the safety and stability of interactive equipment, so it is of great importance to study the control method of rehabilitation robot systems [6–9].

Due to the muscles or nerves not fully recovered, the patient’s affected limb will shake during the rehabilitation training process [10–11], which brings uncertainty to the system and affects the effectiveness of training. Therefore, the design of the rehabilitation system controller should consider not only the rehabilitation robot itself but also the impact of patients on the rehabilitation system and the patients’ recovery condition. These challenges require that the controller design should be improved in robot-assisted rehabilitation [12–14].

Sliding-mode control (SMC) is not sensitive to external interference, making it suitable for designing rehabilitation robot control systems [15–17]. Wege and Hommel [18] proposed a robust sliding-mode controller for hand exoskeleton rehabilitation. Compared with the traditional proportion-integral-derivative (PID) method, this method shows better performance in rehabilitation training. However, the traditional SMC has high requirements for robot models[19,20], which makes it difficult to apply in engineering. To solve and improve the dependence of the mathematical model, Jalali et al. [21] proposed a new model-free adaptive fuzzy sliding-mode controller, which realizes model-free SMC by replacing the equivalent dynamic part in SMC with a fuzzy logic controller. Hu and Tang [22] proposed an adaptive SMC algorithm. The control strategy is essentially a data-driven control method. Therefore, the design of the controller is to use input and output data instead of the control system model. Theoretical analysis proves that the control algorithm can speed up the convergence speed and ensure the stability of the control system. Lee [23] first proposed the concept of proportion-derivative SMC (PD-SMC) in 2004. Ouyang et al. [24] proposed a new PD-SMC control method for translational robots, which is a model-free control law different from standard SMC. The simulation results show that the proposed method can achieve as good results as standard SMC in terms of tracking performance under uncertainty, interference, and varying load conditions.

The above methods have better application potential than traditional SMC, but they only consider the robot itself and do not consider the impact of the humans on the robot. However, in rehabilitation training, it is necessary to consider the patient’s influence on the system and the patient’s health condition [25–26]. Zhang et al. [27] designed an adaptive law related to ankle posture and interaction forces, considering the changes in ankle stiffness during motion, and successfully applied it to the ankle joint robot. Ghannadi et al. [28] proposed a nonlinear model predictive control method for upper limb rehabilitation. Both the established robot model and the two-dimensional upper limb musculoskeletal model consider the influence of contact force. The controller can successfully predict muscle activity of patients with functional impairments while providing rehabilitation therapy for patients. This suggests that we can design the controller by considering the force exerted by patients on the robot, and adjust the control parameters according to the varying force, so as to achieve better human–robot physical interaction.

Impedance control strategy can be used to compensate for interaction forces and disturbances, making it a control strategy that guarantees compliance in human–robot interactions. Using an impedance model to describe the robot’s motion in Cartesian space, Li et al. [8] developed an interactive robot controller capable of understanding the control strategies of human users and optimally responding to their movements. Mokhtari et al. [29,30] applied impedance control to a 7-DOF (degree of freedom) lower limb exoskeleton robot and used the super twisting SMC method to reduce the influence of disturbances and uncertainties on the system while avoiding the chattering phenomenon.

This paper proposes a rehabilitation robot control method called impedance sliding-mode control based on stiffness scheduling (ISMCSS), which can be applied to rehabilitation training with both active and passive modes. A free-model-based SMC strategy is developed to avoid model dependence and reduce the system uncertainty caused by limb shaking. Additionally, the stiffness scheduling rule automatically regulates the impedance parameter of the rehabilitation robot according to the force exerted by the patient on the robot such that the rehabilitation training contributes to catering to the patient’s health condition. The rest of the paper is organized as follows. In the “Design and Implementation of ISMCSS for the Rehabilitation Robot System” section, we give the design, simulation verification, and physical implementation of a rehabilitation robot system. In the “Results and Discussion” section, the proposed method is applied to a real rehabilitation robot, followed by analysis and discussion of the results. In the “Conclusion” section, we conclude this paper.

## Methods

### Rehabilitation robot system

**Fig. 1.**
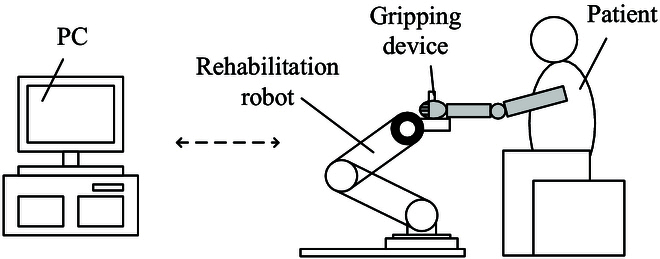
Upper limb rehabilitation robot system.

In this paper, a rehabilitation robot system is designed and applied to upper limb rehabilitation, which consists of a personal computer (PC), a rehabilitation robot, a gripping device, and a user. During rehabilitation training, the PC sends control commands to the robot. The user’s hand grips the gripping device and performs rehabilitation exercises by dragging or following the gripping device. At the same time, the robot acquires the force exerted by the user’s arm at the end of the robot and feeds it back to the control system. The rehabilitation robot system is shown in Fig. 1.

When the robot arm is in contact with the patient, the model of the whole system is equivalent to an impedance system. We use the following impedance model given in the Cartesian space [8], [31]

$$M\ddot{x}+C\overset{\cdot }{x}=u+f$$ (1)

where *x* ∈ ℝ*^m^* represents the actual position of the gripping device at the end of the robot in the Cartesian space. *M* ∈ ℝ^*m*×*m*^ and *C* ∈ ℝ^*m*×*m*^ are desired inertial and damping matrices, respectively. *u* ∈ ℝ*^m^* is the control input in the Cartesian space. *f* ∈ ℝ*^m^* is the interaction force. Because external disturbances and interaction forces have similar effects on the system, there must be disturbances involved when measuring the force exerted by humans on the robot. The external disturbances are also included in *f*.

The kinematics connects the joint space, and the Cartesian space of the rehabilitation robot is described as [31]

$$x=\phi \left(q\right)$$ (2)

where *q* ∈ ℝ*^n^* is the vector of robot joint angle. According to Eq. 2, we have

$$\overset{\cdot }{x}=J\left(q\right)\overset{\cdot }{q},\ddot{x}=J\left(q\right)\ddot{q}+\overset{\cdot }{J}\left(q\right)\overset{\cdot }{q}$$ (3)

where *J* is the Jacobian matrix that maps variables in the joint space to the Cartesian space.

### Control system design

Rehabilitation training is a complex application scenario. Due to human participation, uncertainty will be introduced into the system. For example, during rehabilitation training, due to insufficient recovery, the patient's arm will shake, which in turn affects the control performance.

Since the target behavior is impedance, a basic and effective impedance control law [32] exists when the spring with stiffness matrix *K_d_* and the damper with damping matrix *B_d_* are selected. We add a sliding-mode term to the control law to reduce the instability of the system caused by limb shaking. The sliding surface is designed as follows

$$s=\overset{\cdot }{e}+\beta e$$ (4)

where *e* = *x_d_* − *x*, *x_d_* is the desired position of the robot in the Cartesian space.

The control law is designed as

$$u=K_{d}e+B_{d}\overset{\cdot }{e}+\sigma \text{sign}\left(s\right)$$ (5)

where sign(∗) is a signum function and *σ* is a positive coefficient. This control law can reduce the influence of limb shaking in rehabilitation scenarios.

Patients require different training intensities at different stages of rehabilitation [11,33]. Therefore, the stiffness term *K_d_* of the robot should be adjusted according to the patient’s health conditions to achieve a better rehabilitation training effect.

The patient’s movement can be divided into two steps, namely, retraction and extension. The corresponding relationship between stiffness and contact force is used to build a stiffness-scheduled law.

$$\begin{array}{c} K_{d}\;=\left\{\begin{array}{c} \alpha_{1}\delta f_{trans}+K_{init},\text{if}\;\left|f\right|\leq \;f_{trans}\\ \alpha_{2}\delta f_{\max}+\alpha_{1}\delta f_{trans}+K_{init},\text{if}\;f_{trans}<\left|f\right|\leq \;f_{\max}\\ K_{safe},\text{if}\;f_{\max}<\left|f\right| \end{array}\right. \end{array}$$ (6)

where *α*_1_ and *α*_2_ are the scheduling weights. *δ* is the displacement weight, *δ* = 1/Δ*x*, and Δ*x* represents the change in displacement of the end effector per unit of time during training. *K*_*init*_ is the initial value of the stiffness. *f*_*trans*_ is the threshold set according to different stages of the patient's condition. *f*_*max*_ is the maximum allowable interaction force. *K*_*safe*_ is a small stiffness value. When *f*_*max*_ < |*f*|, *K_d_* should be adjusted to *K*_*safe*_ and then the robot should stop working to ensure patient safety.

If the force exerted by the patient on the robot increases, the stiffness parameter *K_d_* will change accordingly. In active rehabilitation training, it is equivalent to increasing the training difficulty for the patient. In passive rehabilitation training, it is equivalent to the robot’s need to work harder to guide the patient to exercise. In either mode, it will be more beneficial to the patient.

**Fig. 2.**
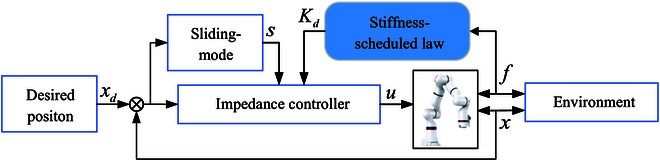
Control system.

The control system diagram is shown in Fig. 2. This design enables the controller to adapt to the different recovery stages of the patient. In addition, this control law can reduce the influence of limb shaking in rehabilitation scenarios. The stability of the proposed controller is confirmed below.

The auxiliary vector *u_d_* is designed as follows

$$u_{d}=M{\ddot{x}}_{d}+C{\overset{\cdot }{x}}_{d}-f$$ (7)

Let Eq. 7 minus Eq. 1, we can get

$$M\ddot{e}+C\overset{\cdot }{e}=u_{d}-u$$ (8)

We demonstrate the stability of the proposed method using the Lyapunov function

$$V=\frac{1}{2}\left[e^{T}\;{\overset{\cdot }{e}}^{T}\right]L\left[\begin{array}{c} e\\ \overset{\cdot }{e} \end{array}\right]$$ (9)

where *L* is a designed symmetric positive definite matrix, when *B* is a semi-positive definite matrix. $L=\left[\begin{array}{cc} B_{d} & M\\ M & \frac{1}{\beta }M \end{array}\right]$.

Deriving the Lyapunov function concerning the time, we can get

$$\overset{\cdot }{V}=\left[\begin{array}{cc} e^{T} & {\overset{\cdot }{e}}^{T} \end{array}\right]\left[\begin{array}{cc} B_{d} & M\\ M & \frac{1}{\beta }M \end{array}\right]\left[\begin{array}{c} \overset{\cdot }{e}\\ \ddot{e} \end{array}\right]$$ (10)

Equation 10 can be rewritten as

$$\begin{array}{c} \begin{array}{l} \overset{\cdot }{V}=e^{T}B_{d}\overset{\cdot }{e}-{\overset{\cdot }{e}}^{T}M\overset{\cdot }{e}+e^{T}M\ddot{e}+\frac{1}{\beta }{\overset{\cdot }{e}}^{T}M\ddot{e}\\ \;=(e^{T}+\frac{1}{\beta }{\overset{\cdot }{e}}^{T})\left(u_{d}-K_{d}e-B_{d}\overset{\cdot }{e}-\sigma \text{sign}\left(s\right)-C\overset{\cdot }{e}\right)+e^{T}B_{d}\overset{\cdot }{e}+{\overset{\cdot }{e}}^{T}M\overset{\cdot }{e}\\ \;=-\frac{1}{\beta }s^{T}\left(\sigma \text{sign}\left(s\right)-u_{d}\right)-e^{T}K_{d}e-\frac{1}{\beta }e^{T}K_{d}\overset{\cdot }{e}-e^{T}C\overset{\cdot }{e}-\frac{1}{\beta }{\overset{\cdot }{e}}^{T}B_{d}\overset{\cdot }{e}-\frac{1}{\beta }{\overset{\cdot }{e}}^{T}C\overset{\cdot }{e}+{\overset{\cdot }{e}}^{T}M\overset{\cdot }{e}\\ \;=-\frac{1}{\beta }s^{T}\left(\sigma \text{sign}\left(s\right)-u_{d}\right)-{\overset{\cdot }{e}}^{T}\left(\frac{1}{\beta }K_{d}+C\right)e-{\overset{\cdot }{e}}^{T}\left(\frac{1}{\beta }B_{d}-M+\frac{1}{\beta }C\right)\overset{\cdot }{e}-e^{T}K_{d}e \end{array} \end{array}$$ (11)

Consider the following conditions for the system,

$$\left\{\begin{array}{l} \sigma >\left\|u_{d}\right\|\;\\ B_{d}-\beta M+C\geq 0\\ K_{d}+\beta C\geq 0\; \end{array}\right.$$ (12)

then by Eqs. 11 and 12, it can obtain that

$$\overset{\cdot }{V}\leq 0$$ (13)

i.e., $\overset{\cdot }{V}$ is semi-negative definite.

Hence, under the inequalities of Eq. 12, the system is globally asymptotically stable based on the Lyapunov method.

To reduce the chattering phenomenon of the controller, under the premise of ensuring the control effect, the saturation function $\mathrm{Sat}\left(s\right)={\left(\mathrm{Sat}\left(s_{i}\right)\right)}_{i=1}^{m}$ is designed as follows

$$\mathrm{Sat}\left(s_{i}\right)=\left\{\begin{array}{c} 1,\;s_{i}>\varepsilon \\ s_{i}/\varepsilon ,\;-\varepsilon \leq s_{i}\leq \varepsilon \\ -1,\;s_{i}<-\varepsilon \end{array}\right.$$ (14)

where *ε* is the boundary layer thickness parameter.

Replace the sign function in Eq. 5 with the saturation function and the control law can be rewritten as

$$u=K_{d}e+B_{d}\overset{\cdot }{e}+\sigma \mathrm{Sat}\left(s\right)$$ (15)

where

$$\begin{array}{c} K_{d}=\left\{\begin{array}{c} \alpha_{1}\delta f_{trans}+K_{init},\text{if}\;\left|f\right|\leq \;f_{trans}\\ \alpha_{2}\delta f_{max}+\alpha_{1}\delta f_{trans}+K_{init},\text{if}\;f_{trans}<\left|f\right|\leq \;f_{max}\\ K_{safe},\text{if}\;f_{max}<\left|f\right| \end{array}\right.. \end{array}$$

### Simulation verification

In this paper, we conducted simulations on a 2-DOF serial planar robotic arm under two sets of external input forces, applying fixed stiffness impedance control (FI), variable stiffness impedance control (VI), and the proposed method ISMCSS separately. By comparing and analyzing the simulation results, we verified the superiority of the proposed control method.

The position of the robot end effector is a two-dimensional matrix *x* = [*x*_1_ *x*_2_]^T^, where *x*_1_ and *x*_2_ represent the position of the robot end effector in the *X* and *Y* directions. Set impedance model inertia and damping as *M* = 6N/m/s^2^,*C* =  −0.2N/m/s. The desired position is set to ${\left[\text{cos}\left(\pi t/3\right)\;+\;\text{cos}\left(\pi t/6\right)\;\text{sin}\left(\pi t/3\right)\;+\;\text{sin}\left(\pi t/6\right)\right]}^{T}m$. In the FI experiment, the stiffness *K_d_* is set to 500 N/m. In the VI and ISMCSS experiments, the moving speed is set as 0.02m/s, ∆*x* = 0.02m, *α*_1_ = 0.8, *α*_2_ = 0.04, *f*_*trans*_ = 10N, *f*_*max*_ = 25N, the initial stiffness *K*_*init*_ = 100N/m, *K*_*safe*_ = 20N/m, and the damping *B*_*d*_ is set as 5N/m/s. That is, when the contact force exceeds 10 N, the stiffness value changes from the initial value of 500 to 1000; when the contact force is less than 10 N, the stiffness value becomes 500. 

The simulation consists of two parts. In the first part, a stable and continuously varying force is applied to the robotic arm to simulate normal rehabilitation movement. In the second part, a force with disturbances is applied to simulate limb shaking. Focusing on the motion of the robotic arm in the *Y* direction, comparative analyses were conducted on position tracking and tracking errors.

**Fig. 3.**
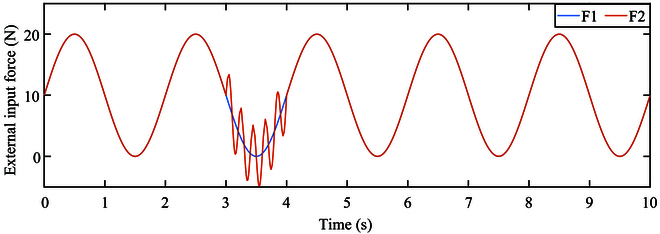
External input force.

The simulated contact force applied to the robot in the *Y* direction is represented by a sinusoidal function 10 + 10 sin (*πt*). In the second part, an additional sinusoidal function 5 sin (10*πt*) is superimposed within the time interval of 3 to 4 s, as shown in Fig. 3. 

**Fig. 4.**
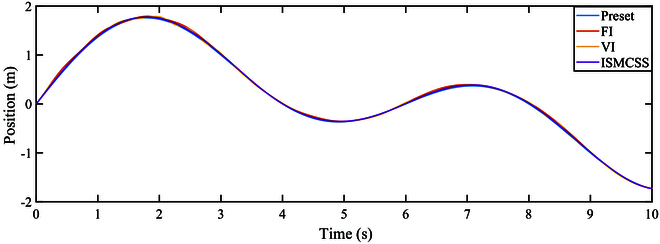
Position tracking under F1.

**Fig. 5.**
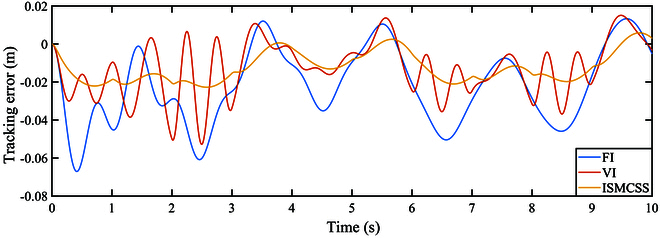
Tracking error under F1.

**Fig. 6.**
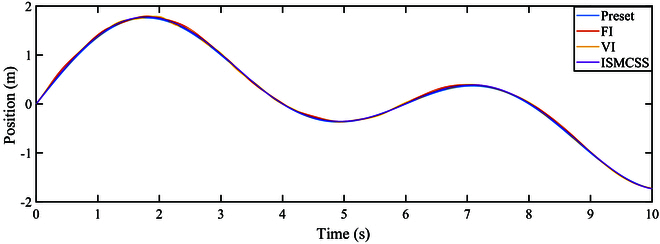
Position tracking under F2.

**Fig. 7.**
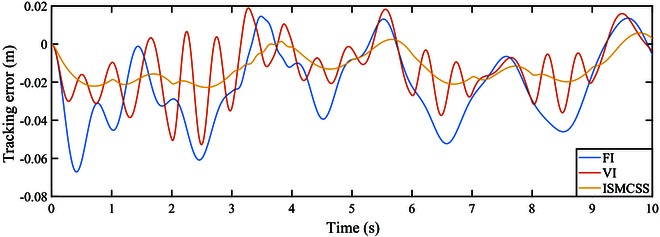
Tracking error under F2.

As indicated by Figs. 4 and 5, under continuously varying contact forces, all three methods perform well in tracking the target position. The tracking error of ISMCSS is smaller than that of FI and VI methods. Figures 6 and 7 show that in the presence of disturbances, all three methods exhibit good tracking of the target position. Still, the tracking error of ISMCSS is smaller than that of FI and VI methods.

Comparing Fig. 7 with Fig. 5, we can find that all three methods exhibit fluctuations in tracking errors during the time intervals when disturbances occur. ISMCSS shows almost identical tracking errors during other time periods, while FI and VI still exhibit fluctuations in tracking errors within 1 s after the disturbance disappears. This indicates that disturbances have varying degrees of impact on the system.

Among the three methods, ISMCSS shows the smallest tracking error, with the least fluctuation in the presence of disturbances. This indicates that the proposed ISMCSS method exhibits excellent tracking performance and is less sensitive to disturbances.

### Experimental process

#### Experimental platform

**Fig. 8.**
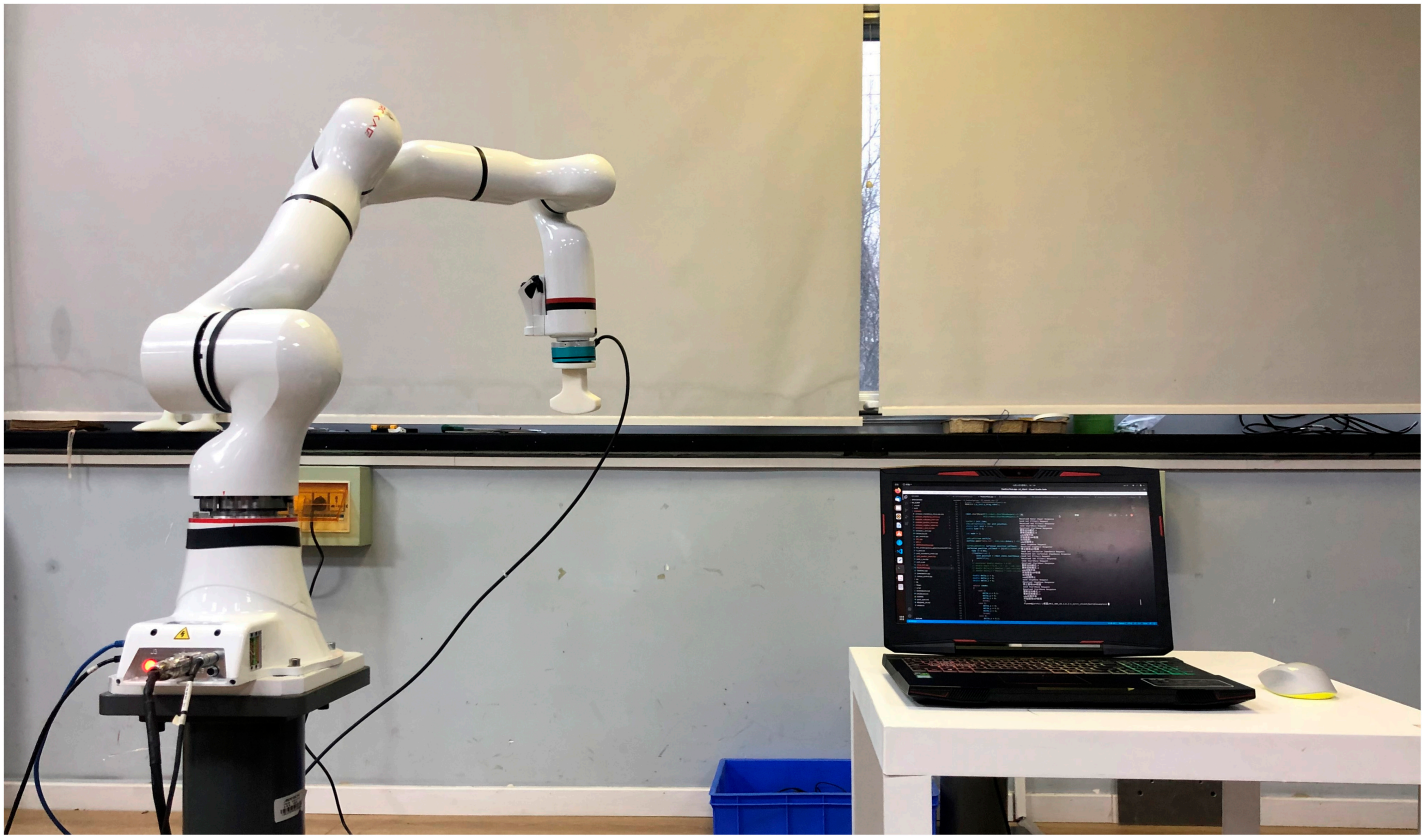
Experiment platform.

The ROKAE Xmate3 robot is used as the main hardware platform for rehabilitation training in this research. The standard Xmate3 robot is a 7-DOF highly dexterous manipulator. In this paper, rehabilitation training is applied to participants’ upper limbs. A recovery device that can be grasped by participants is designed and installed at the end of the robot. Upper-limb rehabilitation experimental setup consists of a robot, a gripping device, and an external PC, as shown in Fig. 8. Depending on the participant’s condition, the participant’s hands can grasp the gripping device positively or can be fixed at the gripping device passively. Then, the participants’ arm could follow the robot to perform various rehabilitation exercises under different training modes.

**Fig. 9.**
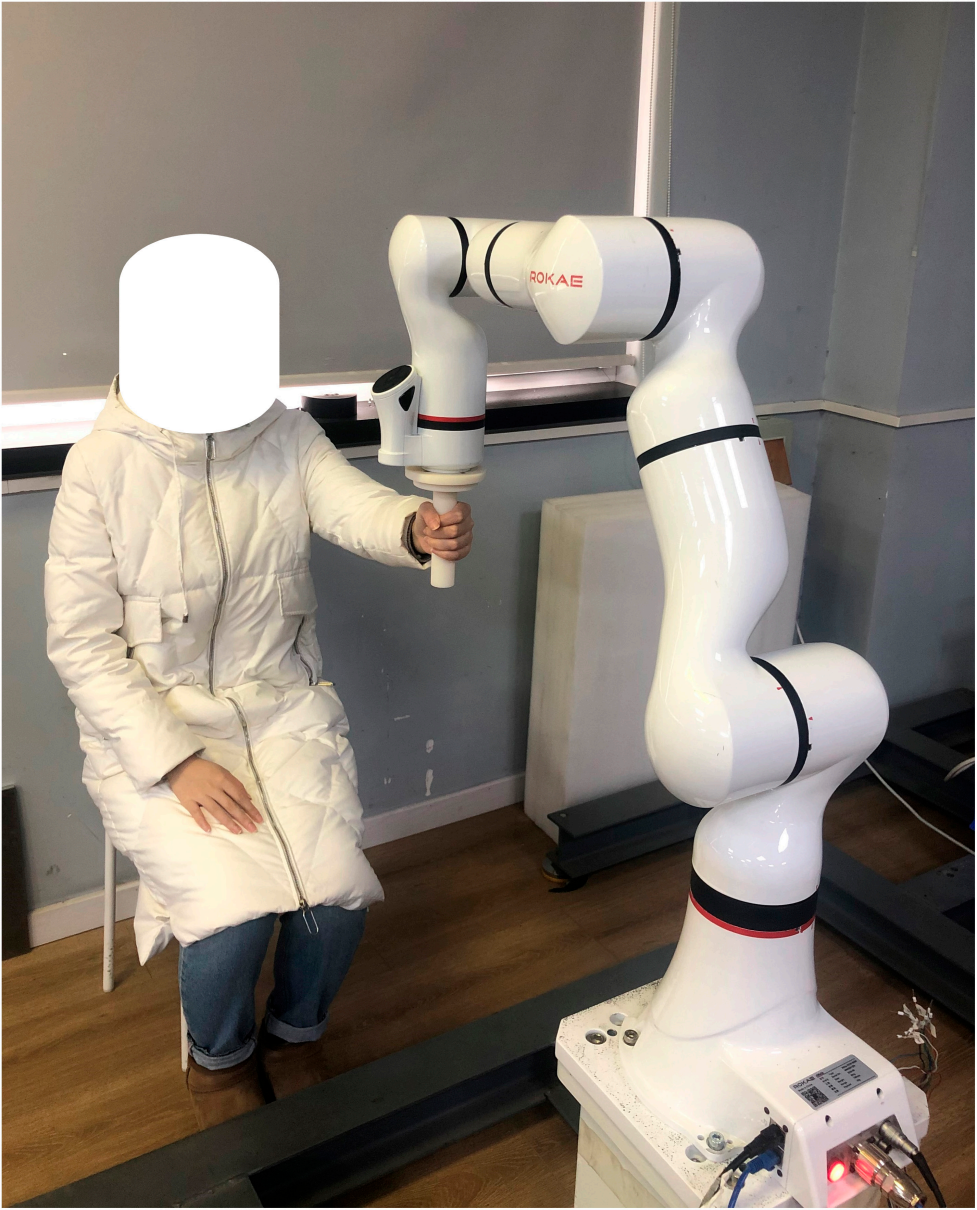
Rehabilitation training scene.

Ten healthy human participants without known sensorimotor impairment participated in the experiment. Before the formal experiment, participants will undergo practice sessions. This pretraining was designed to minimize any potential learning curve effects and enable participants to respond naturally to the robot's actions during the formal experiments. It also helps participants develop a better understanding of various force states and achieve simulations that meet experimental requirements. In formal rehabilitation training experiments, each participant should be set to a sitting position, try to move the “affected” limb, and keep the rest of the body motionless. This paper focuses on feasible human–robot interaction control methods that consider human influence and conducts experiments on healthy participants. The rehabilitation training scene is shown in Fig. 9.

#### Experimental design

This paper designs a rehabilitation exercise for the upper limb of participants. The designed reciprocating linear motion meets the requirements of simple movements and multiple repetitions in rehabilitation training.

**Fig. 10.**
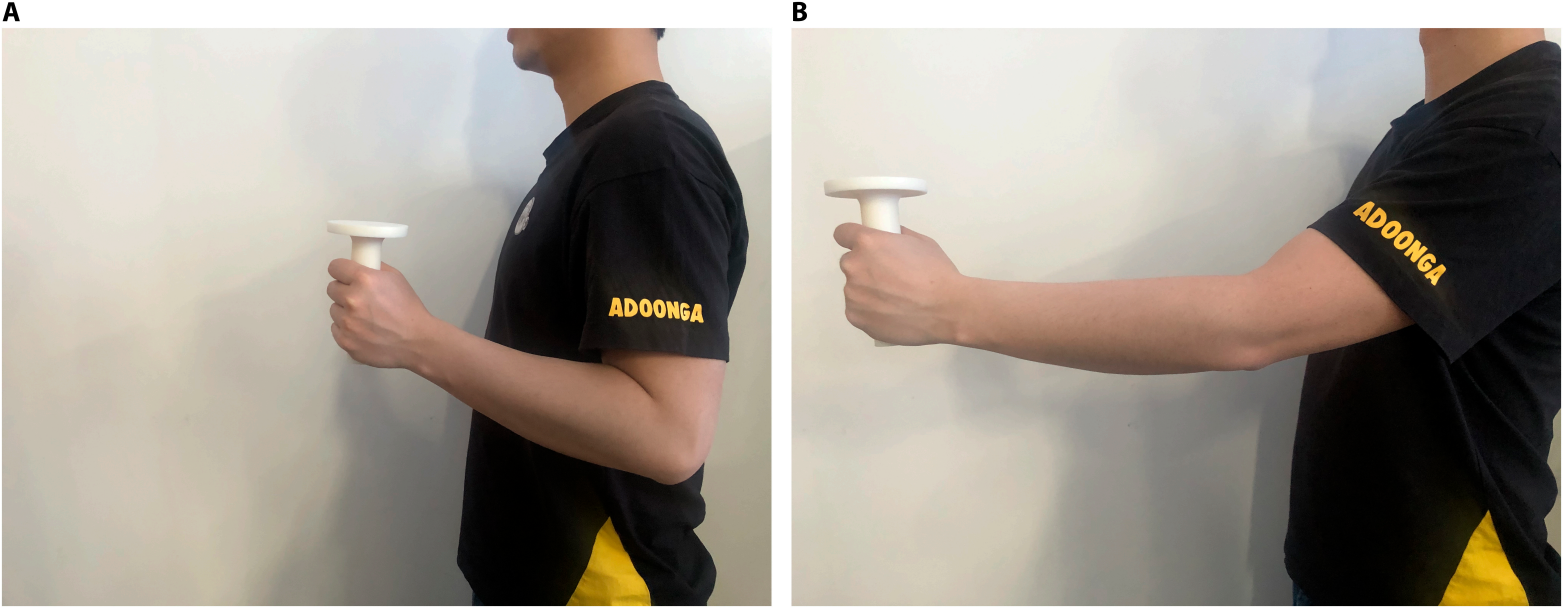
Training action. (A) Retraction. (B) Extension.

In rehabilitation training, the participant raises the forearm so that it is parallel to the ground and then bends the arm so that the upper and lower arms are perpendicular to each other, grabs the gripping device, and performs reciprocating linear motion along with the robot. The training trajectory of the hand is approximately a straight line, and the range is 20 cm, about the length of an upper arm. This action can effectively exercise the patient’s arm muscles. The action is simple and user-friendly. The participant can have a certain sense of control and security during training. The diagram of the training action is shown in Fig. 10.

**Fig. 11.**
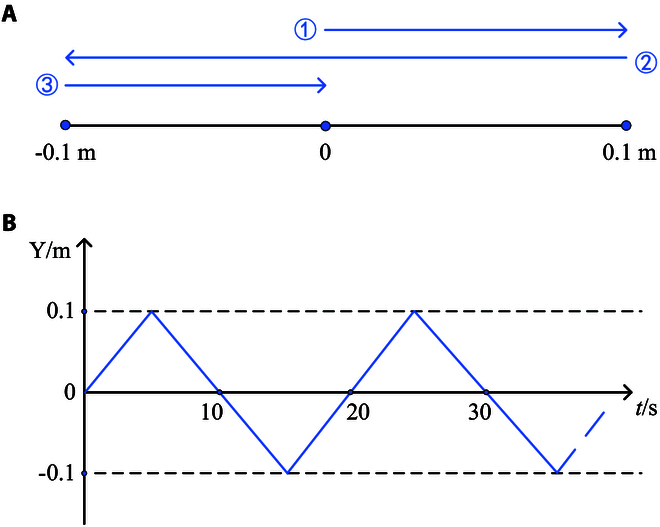
Motion trajectory. (A) Rehabilitation training trajectory diagram. (B) Expected position diagram.

The participant's hand movement diagram is shown in Fig. 11. An extension-retraction cycle is completed by the sequence and direction in Fig. 11A. Figure 11B shows the expected position of the participant's hand and the end of the robot over time.

The experiment set the rehabilitation movement from the starting point, along a fixed direction. The patient’s hand moves to 0.1 m along the positive direction and then changes the direction to −0.1 m for reciprocating motion. The parameter values in Eq. 6 need to be selected according to different groups of people. In the experiment of this paper, the moving speed is set to 0.02m/s, Δ*x* = 0.02m, *α*_1_ = 1, *α*_2_ = 0.125, *f*_*trans*_ = 10N, *f*_*max*_ = 80N, the initial stiffness *K*_*init*_ = 500N/m, *K_safe_* = 20N/m, and the damping *B_d_* = 1N/m/s.

During the experiment, when the contact force exceeds the safe value, the training should not be continued to ensure the safety of the participants. The above experiments were carried out using the method proposed in this paper, and the results were analyzed and discussed as follows.

## Results and Discussion

### Adaptability: Assisted rehabilitation experiments for different participants

The rehabilitation system designed in this paper is aimed at patients with sensorimotor impairment after fracture or stroke. These patients may have trouble moving, limb shaking, and other phenomena. We correlate the possible behaviors of the patient with the effects of these behaviors on the rehabilitation system. When the patient autonomously follows the robot, it corresponds to minimal resistance experienced by the robot. Similarly, when the patient completes rehabilitation training while following the robot, it signifies that the robot encounters stable resistance. If the patient faces difficulty in following the robot during rehabilitation training, it indicates an increased resistance being applied to the robot. Additionally, limb shaking exhibited by the patient during rehabilitation training corresponds to additional interference imposed on the robot. The experiment was conducted by the 10 participants described in the “Experimental design” section, who performed simulations based on the above description. This part verifies the adaptability of the proposed controller through the passive rehabilitation experiments of 10 participants.

**Fig. 12.**
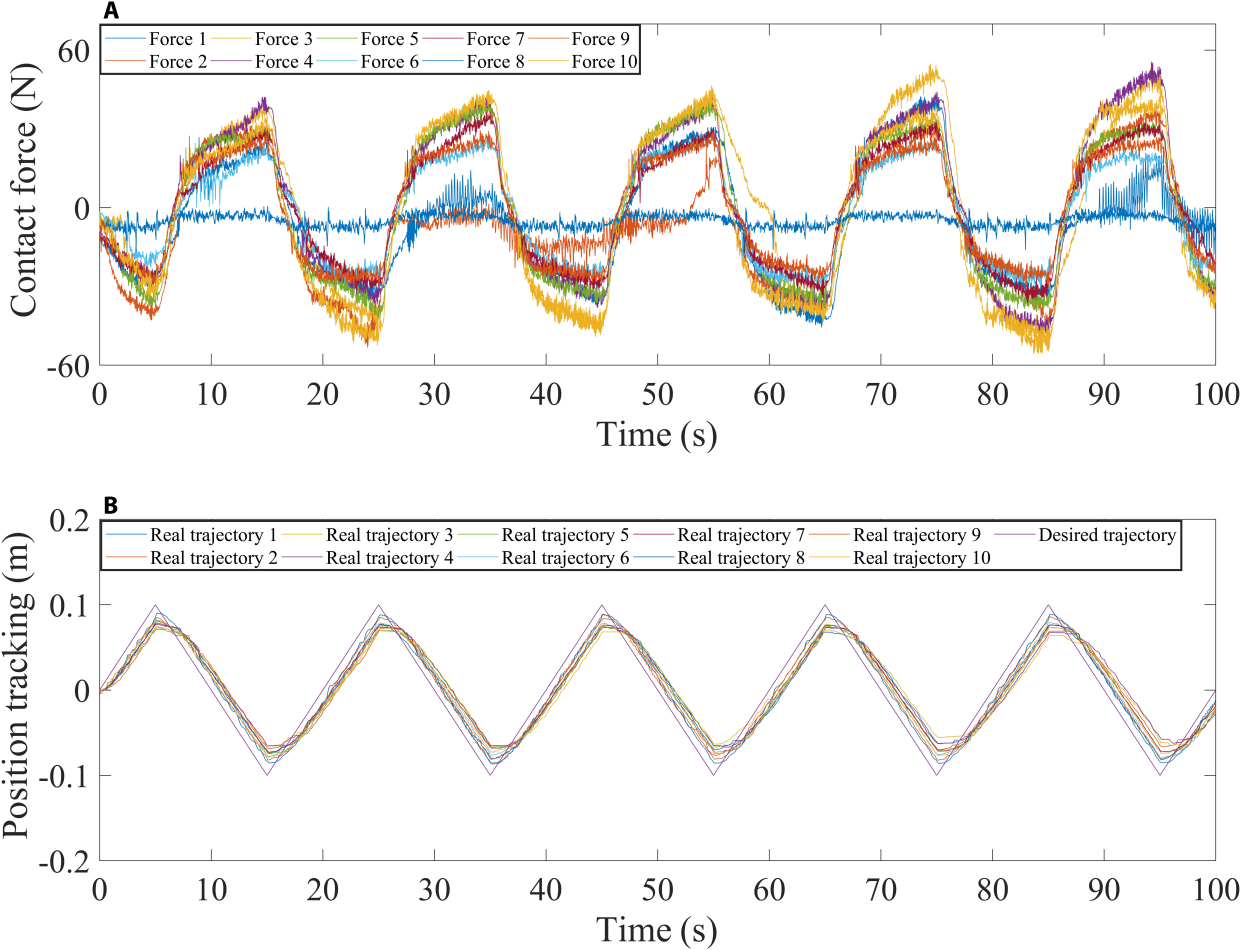
Results from 10 participants. (A) Contact force. (B) Position tracking.

Figure 12A shows the contact forces 1 to 10 generated by participants 1 to 10. During the movement, participants 1 and 2 exhibited noticeable shaking. The data from forces 3, 4, and 10 suggest that participant 3, 4, and 10’s sensorimotor impairments led to significant resistance against the robot's movements. Participants 5, 6, 7, and 9 applied steadily varying resistance to the end of the robotic arm, indicating that they could perform normal rehabilitation training. Participant 8 exerted less force on the end of the robotic arm, indicating that he had the ability to move with the robot.

As shown in Fig. 12B, all participants have completed the rehabilitation training, and during the passive rehabilitation training, all participants move within the prescribed movement range without overshooting. However, the movement of all participants has a certain time delay, and it is analyzed that all participants need a certain reaction time to follow the movement of the robot, and the resistance forces exerted by participants to the robot will also cause the movement delay.

The above analysis indicates that the proposed controller can adapt to different people and different conditions so that humans and robots can complete rehabilitation training.

### Versatility: Rehabilitation experiments are completed in different modes

Experiments were conducted in two rehabilitation modes: active rehabilitation training mode and passive rehabilitation training mode [34]. In the active rehabilitation training mode, the patient actively completes the rehabilitation exercise. If the patient cannot complete the task, the robot can assist and guide the patient’s hand. In the passive rehabilitation training mode, three situations are introduced for discussion: (a) Participants apply stable resistance to the robot. (b) Participants exert interference force on the robot. (c) Participants exert extreme force on the robot. The experimental results of a representative participant are selected for discussion and analysis in this part.

#### Active rehabilitation training

During active rehabilitation training, the patient already has a certain degree of autonomous movement ability and can actively apply force to the end of the robot during the rehabilitation exercise. Therefore, the participant applies active and stable assistance to the end of the robot by simulating the patient undergoing active rehabilitation training.

**Fig. 13.**
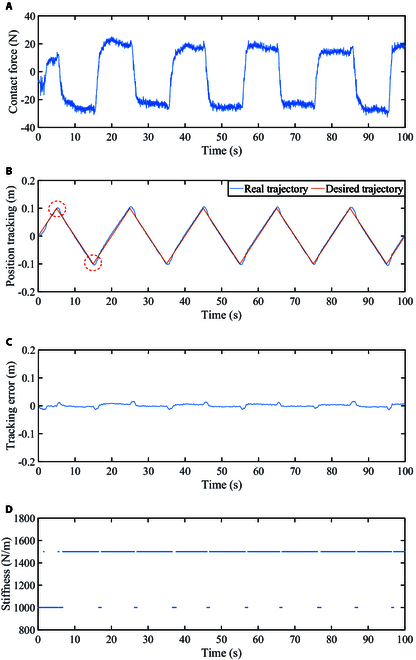
Active mode. (A) Contact force. (B) Position tracking. (C) Tracking error. (D) Stiffness variation diagram.

The obtained contact force diagram is shown in Fig. 13A. The contact force is approximately between −30 N and 25 N, and it changes periodically with the reciprocating motion of the arm. Figure 13B depicts position tracking for this rehabilitation training. Under the proposed method, the participant can complete the preset rehabilitation training together with the robot. As can be seen from the figure, the real positions are very close to the expected positions during the extension and contraction phases but deviate from the expected position during the switching direction phase (see the area marked by the red dotted circle). The analysis is that in the active mode, the participants have good activity and there is motion inertia, which leads to the trajectory deviation when the direction of motion is switched.

The tracking error diagram of this rehabilitation training is shown in Fig. 13C. The tracking error is between −0.015 and 0.02, and fluctuates around 0, showing periodic changes. Due to the noise in the collected force, we make the stiffness change with the value of the filtered force, as is the case in all experiments in this paper. The changes in stiffness are illustrated in Fig. 13D.

From the above analysis, it can be seen that under the proposed control method, the participant can move well according to the preset trajectory, and the system remains stable. In the middle and late stages of the patient's rehabilitation, the rehabilitation robot can assist the patient in completing the rehabilitation training task through the proposed method.

#### Passive rehabilitation training

In passive rehabilitation training, the participant follows the robot to move, and can passively apply force to the end of the robot during the rehabilitation exercise.

**Fig. 14.**
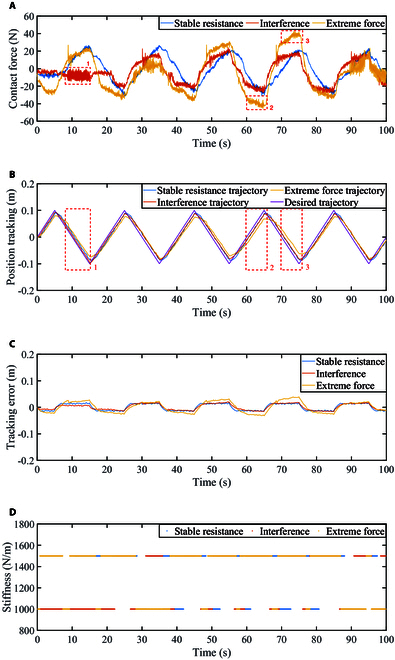
Passive mode. (A) Contact force. (B) Position tracking. (C) Tracking error. (D) Stiffness variation diagram.

Figure 14A shows the change of contact forces in the three sets of passive rehabilitation training. In the stable resistance experiment, the participant can follow the robot to complete the rehabilitation training without arm shaking and weakness. From the stable resistance curve in Fig. 14A, it can be seen that the resistance changes periodically. The interference contact force curve shows obvious fluctuations, indicating that the participant's arm is constantly shaking, such as the area marked by the red dashed rectangle 1. During the rest time, the contact force is cyclically changed, the same as the stable resistance. The extreme force curve refers to the extreme force exerted by the participant on the end of the robot due to excessive muscle tension so that the participant cannot follow the robot to complete the expected movement. It can be seen from the red dashed rectangles 2 and 3 in Fig. 14A.

The arm movements are depicted in Fig. 14B. The position tracking results of the three groups of experiments have time delays in different degrees. This suggests that participants had some difficulty keeping up with the training, which affected the speed of response. When switching direction, the tracking errors were greater than in extension or retraction, indicating that the participants could not fully reach the preset position.

The forces marked in the red dashed rectangles 1 to 3 in Fig. 14A correspond to the motion trajectories marked in the red dashed rectangles 1 to 3 in Fig. 14B, respectively. From rectangle 1, it can be seen that the participant can still complete the following motion under arm shaking, and the following effect is better than the other two situations, indicating that the participant can follow the robot to complete the preset rehabilitation training. As can be seen from rectangles 2 and 3, when the resistance is large, the following effect is not good, and there is a large delay and error, indicating that the participant’s motion range is limited and smaller than the preset motion range.

Tracking accuracy results are shown in Fig. 14C. It can be seen that the tracking accuracy is related to the force. When the force is small, although it is accompanied by shaking, the tracking accuracy is still good. And the tracking error becomes larger with the emergence of extreme force. The three sets of stiffness values change with the variation of the three sets of contact forces, as shown in Fig. 14D.

By analyzing the results of the three sets of experiments, the following conclusions can be drawn. The proposed method can be stably applied to the designed upper limb rehabilitation robot system under stable resistance, disturbance, and extreme force conditions. Accurate position tracking and system stability can be achieved in a stable resistance situation. In the presence of interference, it can achieve good position tracking and maintain system stability, and greatly reduce the impact of limb shaking on the robot system during the rehabilitation training process. When extreme force occurs, poor position tracking helps prevent harm to the participants and ensures their safety.

In the early stage of the patient’s rehabilitation, the rehabilitation robot can guide the patient to complete the rehabilitation training task through the proposed method.

## Conclusion

In this paper, an impedance sliding-mode control method based on stiffness-scheduled law is proposed and successfully applied to an upper limb rehabilitation robot system. Both patient–robot interaction forces and patients’ health conditions have been considered in the controller design of the rehabilitation robot system. The proposed method uses an SMC strategy to reduce the influence of the patient’s affected limb shaking and uses the designed stiffness-scheduled law to select a suitable control law parameter to achieve more personalized rehabilitation training. Through multi-person multi-mode experiments, it is verified that the proposed rehabilitation system can maintain stability in the face of participants’ shaking and extreme force, and assist or guide participants to carry out rehabilitation training.

## Data Availability

Research data used to support the findings of this study are available from the corresponding author upon request.
